# Assessing the facilitators and barriers of interdisciplinary team working in primary care using normalisation process theory: An integrative review

**DOI:** 10.1371/journal.pone.0177026

**Published:** 2017-05-18

**Authors:** Pauline O’Reilly, Siew Hwa Lee, Madeleine O’Sullivan, Walter Cullen, Catriona Kennedy, Anne MacFarlane

**Affiliations:** 1 Department of Nursing and Midwifery, Faculty of Education and Health Sciences, University of Limerick, Limerick, Republic of Ireland; 2 School of Nursing and Midwifery, Robert Gordon University, Aberdeen, United Kingdom; 3 Graduate Entry Medical School (GEMS), Faculty of Education and Health Sciences & Health Research Institute, University of Limerick, Limerick, Republic of Ireland; 4 School of Medicine and Medical Sciences, University College Dublin, Dublin, Republic of Ireland; University of Stirling, UNITED KINGDOM

## Abstract

**Background:**

Interdisciplinary team working is of paramount importance in the reform of primary care in order to provide cost-effective and comprehensive care. However, international research shows that it is not routine practice in many healthcare jurisdictions. It is imperative to understand levers and barriers to the implementation process. This review examines interdisciplinary team working in practice, in primary care, from the perspective of service providers and analyses 1 barriers and facilitators to implementation of interdisciplinary teams in primary care and 2 the main research gaps.

**Methods and findings:**

An integrative review following the PRISMA guidelines was conducted. Following a search of 10 international databases, 8,827 titles were screened for relevance and 49 met the criteria. Quality of evidence was appraised using predetermined criteria. Data were analysed following the principles of framework analysis using Normalisation Process Theory (NPT), which has four constructs: sense making, enrolment, enactment, and appraisal. The literature is dominated by a focus on interdisciplinary working between physicians and nurses. There is a dearth of evidence about all NPT constructs apart from enactment. Physicians play a key role in encouraging the enrolment of others in primary care team working and in enabling effective divisions of labour in the team. The experience of interdisciplinary working emerged as a lever for its implementation, particularly where communication and respect were strong between professionals.

**Conclusion:**

A key lever for interdisciplinary team working in primary care is to get professionals working together and to learn from each other in practice. However, the evidence base is limited as it does not reflect the experiences of all primary care professionals and it is primarily about the enactment of team working. We need to know much more about the experiences of the full network of primary care professionals regarding all aspects of implementation work.

**Systematic review registration:**

International Prospective Register of Systematic Reviews PROSPERO 2015: CRD42015019362.

## Introduction

Internationally health care delivery faces many challenges, such as changing demographics and related health care needs, persisting health inequalities, and increasing financial constraints. Health care policies are focusing on optimising health care provision with an emphasis on interrelated issues such as improved patient outcomes, increased effectiveness, reduced costs and integrated care delivery [1–3]. In this context, the World Health Organisation (WHO) emphasises that interdisciplinary team working in primary care is of paramount importance in the reform of health care [4].

The current review employed the term “interdisciplinary team” as a generic term of reference for health care teams which include a range of health service workers, both professionals and non-professionals, with the majority being from professional groups. Across health care systems, policies promote interdisciplinary working in primary care and associate it with improving the quality and efficiency of health care with positive impact on patients and providers alike [5–9]. These “top down” policies encourage the development of interdisciplinary working in primary care settings. While there is evidence of progress with the implementation of these policies in countries such as the United Kingdom (UK) [10], there is less progress in other countries such as the United States of America (USA) [11] and Ireland [12]. Therefore, it is important to advance the evidence base about the implementation of interdisciplinary work in primary care to fully understand, and close, this policy–practice gap.

Research to date has shown that this kind of reform is acknowledged as a substantial organisational change process and that staff require support during the process [13]. A prerequisite for supporting staff is to understand how they think about innovation and organisational change processes and what happens when they try to enact it in daily practice [14]. Mickan et al. [15] studied interdisciplinary practice in primary care across 10 countries and collated findings from health professionals about their needs for team working. They found that service providers emphasised the importance of having clear policies in place about interdisciplinary team working, clarity about each other’s expectations, regular team meetings, open communication and a clear focus on patient care. Participants across many of the 10 countries reported that, in practice, they have experiences of poor communication and interpersonal conflicts as barriers to change. Supportive legislation and governance models, and committed leadership were viewed as being facilitators.

Other studies have identified similar findings about these barriers and facilitators in practice [16–22]. In addition, the benefits of co-located professionals to facilitate interprofessional team working have been documented [16, 17, 22]. There are also “deeper” barriers relating to professional socialisation (described in the work of sociologist Freidson [23]). In some cases, some members of the team may not want to change the way they were initially socialised into their profession, particularly physicians [24].

To summarise, while “top down” policies promoting interdisciplinary team working in primary care are evident across international settings, there are challenges with the reform process to make this a routine and normalised way of working. There is a body of knowledge about the experiences and problems that primary care professionals have when they attempt to work together across disciplines. Furthermore, some reviews have synthesised the evidence about specific issues such as physician and nurse team working [25] and there have been studies about implementation of team working in primary care in specific national settings [26] or across settings [27]. However, there has been no theoretical synthesis of *all* primary care professionals’ experiences of team working in practice and across countries with a view to identifying overarching and common levers and barriers to implementation.

We have addressed this problem by employing a theoretical framework to review international literature about interdisciplinary team working in practice. We have employed Normalisation Process Theory (NPT) as a heuristic device to “think through” descriptions of health care practices from a variety of primary care providers and countries in order to identify potentially generalisable levers and barriers to implementation. Developed by May and Finch [28], NPT is a framework that can be applied across health care contexts and topics to explain, and potentially shape, implementation processes [29].

NPT has four constructs that provide a conceptual framework for this process (see Table 1). To the best of our knowledge, this is the first theoretically informed systematic analysis of interdisciplinary team working in primary care.

**Table 1:** Normalisation process theory: Constructs and explanations.

Constructs	Explanation
Coherence	Sense-making: Do stakeholders grasp the concept of an innovative practice?
Enrolment	Engagement: Do stakeholders “buy into” an innovative practice and seek to drive its implementation forward?
Collective Action	Enactment: Can stakeholders enact the new innovation into practice in a real-world setting?
Reflexive Monitoring	Appraisal: Can stakeholders evaluate the impact of innovation and generate ideas for reconfiguring practices to sustain its use over time?

The objective of this review was to examine accounts of interdisciplinary team working in practice in primary care from the perspective of service providers and to analyse: (1) What does the published literature tell us about barriers and facilitators to implementation of interdisciplinary teams in primary care? (2) What, if any, are the main research gaps?

## Methods

The protocol for this study was registered with the International Prospective Register of Systematic Reviews (PROSPERO) CRD42015019362 http://www.crd.york.ac.uk/PROSPERO [30]. This review is reported following the Preferred Reporting Items for Systematic Reviews and Meta-analyses (PRISMA) [31].

Integrative reviews, also known as mixed methods reviews, synthesise evidence from empirical studies using both quantitative and qualitative methods [32]. We used a deductive Framework Analysis approach [33] using NPT (see Table 1). Whilst the majority of the studies were qualitative the integrative review followed a systematic process and was informed by PRISMA and SIGN criteria [31] [34].

### Eligibility criteria

We searched for articles published in English between January 2004 and February 2015. The search strategy is detailed below.

### Search strategy

The search strategy included 10 electronic international databases (Box 1). We piloted the search terms in MEDLINE in order to determine their sensitivity and specificity to the review questions. Two authors (MOS, SL) then screened the titles and abstracts of the piloted results independently and discussed the inclusion and exclusion criteria with all the review team members. Following the pilot we consulted with an Information Specialist (Librarian) and the search terms were adapted to the other databases. Adaptations to the search strategy at this point were inclusion of the search terms *primary health services* and *community health services* in recognition of the variation of terminology used in different countries. The final search terms used included synonyms and Medical Sub-Headings (MeSH) describing primary care, teams and team working (the search string can be found in Box 2).

**Box 1. Summary of searched databases and other sources.**

Databases:

- Cochrane Library (Cochrane Database of Systematic Reviews, Cochrane Central Register of Controlled trials (CENTRAL), Cochrane Methodology Research)
- MEDLINE
- EMBASE
- CINAHL
- PsycINFO
- AMED
- ASSIA
- TRIP
- ISI Web of Science
- Scopus

Unpublished work (grey literature) which is not published in accessible formats or indexed in the academic databases listed below:

- Conference proceedings
- Hand searching articles from reference lists of included studies

Ongoing studies:

- www.who.int/ictrp/en/
- www.anzctr.org.au
- www.clinicaltrials.gov
- www.controlledtrials.com

**Box 2. Search strategy: MEDLINE format.**

1. (MH “Primary Health care+”)
2. “Primary care”
3. “Primary Health Services”
4. “Community care”
5. (MH “Community Health Services”)
6. or/1–5
7. Team*
8. Interdisciplin* or inter-disciplin* or interprofession* or inter-profession* or multidiscipline* or multi-disciplin* or multiprofession* or multi-profession*
9. or/7–8
10. Collaborat* or cooperat* or co-operat*
11. 6 and 9 and 10
12. Limit 11 to year = 2004–2015

### Study selection

We focused on research about interdisciplinary team working in formal, statutory primary care services. We included quantitative and qualitative studies. The majority of the evidence dealing with the subject area was qualitative in nature.

We considered studies that described empirical data about:

1. Interdisciplinary team working within a formal statutory primary care team serving the general population;
2. A team member talking about interdisciplinary working with at least one other professional who is a team member (including general practitioners; nurses; physiotherapists; occupational therapists; social workers; managers and administrative staff; community representatives).

We excluded studies that reported specialist teams established or focused to work in specific areas such as maternal and child health, veterans, mental health, depression and psychiatry. However, articles about team working in primary care for one of these specialist populations were included if the team was established to serve the whole population but was reporting one aspect of its work with a specialist population. We excluded studies whose focus was on the education or training of undergraduates/postgraduate health care professionals in interdisciplinary team working. We excluded systematic reviews, non-empirical studies, commentary, discussion and opinion papers.

### Data selection process

All authors acted as reviewers and worked in pairs to independently screen the titles and abstracts for potentially relevant studies. The pairs then met to discuss their screening results. Queries were brought to team meetings for further discussion until consensus was reached. Potential full text articles were retrieved and screened by two reviewers (MOS, SL). Potentially relevant studies were agreed and divided among the full team of six authors for data extraction.

### Quality appraisal of included studies

A screening and appraisal tool for qualitative papers adapted from Noyes and Popay [35] and Kennedy et al. [36] and a quality assessment tool adapted from the Effective Public Health Practice Project [37] for quantitative studies were used. The quality of evidence for quantitative and qualitative papers was appraised using the Scottish Intercollegiate Guidelines Network (SIGN) criteria [34].

## Data analysis and synthesis

The coding frame for our NPT deductive analysis is shown in Table 2. Relevant data from included papers were electronically coded from PDFs onto a customised data extraction template. Details extracted include the author(s); year; country in which the study was published; journal; study aim; research design; data collection methods; health care professionals involved; study setting; approach to data analysis; quality of study; quality of evidence; strengths and limitations; data about sense making, enrolment, enactment and appraisal; non-NPT data, notes; and key message. The data relating to the NPT constructs in the completed data extraction templates were coded in NVivo 10 [38], one node for each of the four constructs, with annotations and coding decisions recorded for clarity of purpose. To ensure trustworthiness and rigour of the analysis the team then held a two-day data analysis clinic to review coding decisions and reach consensus about any queries that arose. Coding decisions and emerging findings were summarised, recorded and transcribed to serve as a guide for the next stage of analysis. The analysis was completed by the team through a combination of face-to-face and electronic communication.

**Table 2:** Normalisation process theory: Coding frame for integrative review of interdisciplinary team working in primary care.

Sense making	Enrolment	Enactment	Appraisal
• How is the idea of interdisciplinary team working understood by participants? • How do they compare it with existing practices–is it regarded as something usual or novel? • Do all participants see its potential value? • Can participants from individual professional groups make sense of the work that interdisciplinary team work would create for them?	• Do participants think it is right for them to be involved in interdisciplinary team working? • Can they drive this way of working forward? • How and why do the participants come to take part in an interdisciplinary team? • What keeps them motivated to continue taking part?	• What resources (financial, policy, staffing) are available to support interdisciplinary team working? • Do participants have appropriate skills and clarity about effective divisions of labour? • Do participants have trust and confidence in their own work and the work of other colleagues in the team? • How are team working activities organised and structured and do they “fit” with existing routines?	• Can participants evaluate the impact of interdisciplinary team working, using informal or formal evaluations to ascertain its impact? • Do participants from individual professional groups think it is worthwhile for them? • Do participants across professional groups agree about its value and impact? • Can existing practices be changed to sustain team working?

## Results

### Study selection

**Fig 1:**
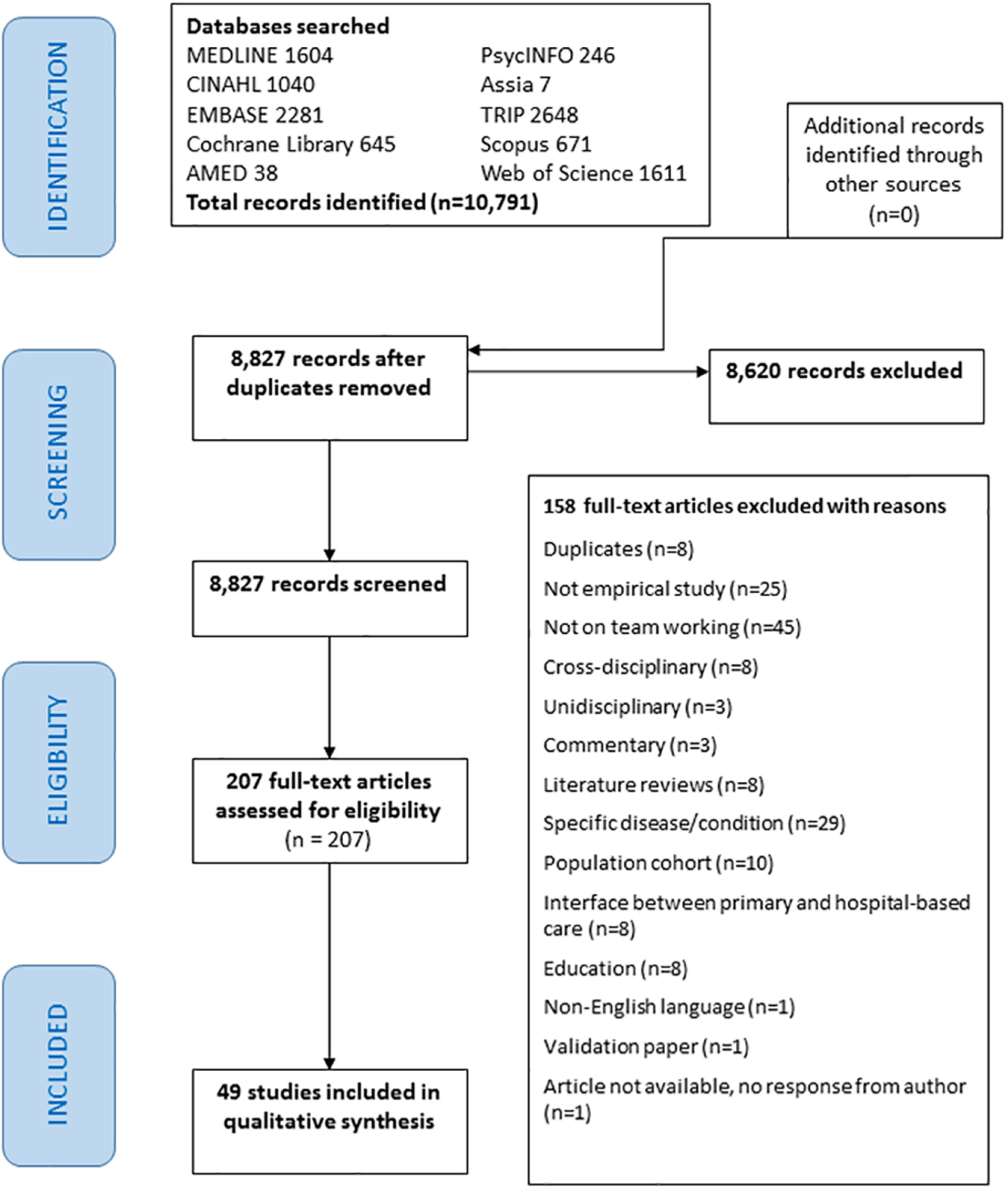
PRISMA flow diagram [31].

The initial search yielded 10,791 titles from 10 databases. A total of 8,827 titles and abstracts were screened after de-duplication. At first screening of potentially relevant full texts of publications, 207 were assessed for eligibility and 49 studies were included in our final review (Fig 1). A total of 158 papers were excluded as they did not meet our inclusion criteria: for example, we excluded studies on specific disease/condition outcomes, discussion and opinion papers, commentaries, literature reviews, interface between primary and hospital-based care, population cohort studies, education for undergraduates/postgraduates and non-empirical studies. No studies were identified from the reference lists of the included studies, grey literature or ongoing studies. The author of one of the papers was contacted to establish if the study had been completed but we did not receive a response.

### Characteristics of included studies

The included studies were: qualitative (*n* = 39) [39–77], quantitative (*n* = 2) [78, 79], and mixed method (*n* = 8) [80–87] (S1 Table). The studies were from 11 countries and most of the studies were conducted in Canada (*n* = 17) [45, 51–53, 55, 57, 63, 65, 66, 71, 78–80, 82–84, 87], followed by the USA (*n* = 12) [40, 43, 44, 47, 49, 56, 58, 72, 76, 77, 85, 86], the UK (*n* = 7) [39, 48, 59, 64, 68, 70, 81], Australia (*n* = 4) [42, 73–75], New Zealand (*n* = 2) [41, 62], Sweden (*n* = 2) [54, 67], France (*n* = 1) [50], Spain (*n* = 1) [46], Netherlands (*n* = 1) [60], Brazil (*n* = 1) [61] and Republic of South Africa (*n* = 1) [69]. Thirty-two different primary care professionals participated in these studies, with most of the data relating to General Practitioners/Family Physicians (42 papers), nurses (35 papers), pharmacists (14 papers) and administrative staff (11 papers) (Table 3). There were fewer than 10 papers with data about the other health professionals. Only one paper referred to the involvement of community health workers in the interdisciplinary network [61].

**Table 3:** Breakdown of professional groups represented in the review papers, in alphabetical order.

Health Professional *n* = 32	Number of papers in which professional is mentioned
ACS (Community Health Agent, Brazil)	1
Administration staff (e.g. receptionist, filing clerks)	11
Biomedical Analyst	1
Case Managers	2
Chiropractors	4
Diabetic Educators	1
Dieticians	5
Exercise Physiologists	2
General Practitioners/Family Practitioners	42
Health Promoters	1
Health Visitor/Assistant	2
Informatics	1
Internal Medicine	1
Massage Therapist	1
Mental Health Worker	1
Midwife	1
Nurses	35
Obstetrician/Gynaecologist	2
Occupational Therapists	3
Patient Educators	2
Pharmacist/Community Pharmacist	14
Physician Assistants	3
Physiotherapists	6
Podiatrist	1
Practice Managers	3
Psychiatrists	2
Psychologists	4
Respiratory Therapists	2
Senior Primary Care Team Medical Directors	3
Social Workers	9
Specialist Palliative Care	1
Speech and Language Therapists	3

Fifteen papers were based on co-located teams [43, 48, 49, 53, 54, 56, 58, 65, 70, 71, 75, 83–85, 87] and 16 were from settings where the team is made up of some co-located professionals who are working with others in different sites [40, 42, 45, 46, 60, 64, 66, 68, 69, 73, 74, 80–82, 86]. Details about the physical infrastructure of the team being studied were not explicit in 18 papers [39, 41, 44, 47, 50–52, 55, 57, 61–63, 67, 72, 76–79].

### Quality of included studies

We used the SIGN levels of evidence to assess the quality of the 49 papers. The SIGN scoring system ranges from a 1++ score for high-quality systematic reviews or randomised controlled trials (RCTs) with a low risk of bias to a score of 4 for expert opinion-based evidence. Of the 49 included studies 48 were rated as Level 3. One mixed method study which comprised an RCT was rated as SIGN 1 [84].

### Sense making

Seventeen papers reported on the construct of sense making [39, 42, 44–46, 48, 57, 60, 63, 65–67, 70, 72, 80, 82, 87]. Eight papers were from Canada, three from the UK, two from the USA, and one from each of Sweden, Spain, The Netherlands and Australia.

The findings showed that interdisciplinary team working was typically viewed as a positive idea and that there was a good understanding across health care professional groups as to what working within a team should be like [39, 44, 45, 48, 57, 60, 63, 65–67, 72, 80, 82, 87]. Team work was typically associated with collaboration with different disciplines in the delivery of care to enhance patient outcomes [39, 48, 57, 60, 63, 65–67, 72, 82, 87]. It did not mean being subsumed into a single organisational or professional framework where the team was driven by one profession or agenda [57, 60].

Interdisciplinary team working was reported as routine practice by health professionals in primary care in only four papers, from three countries: UK (*n* = 2); USA (*n* = 1); Sweden (*n* = 1) [39, 44, 48, 67]. Health professionals from across disciplines can see the potential value of interdisciplinary working both for their own experience as professionals and for patients’ experiences and outcomes, with a strong emphasis on the latter [45, 46, 63, 65–67, 72, 82, 87].

*The benefactor is the client*, *having a multidisciplinary collaboration to share the goal of keeping the person viable–living in their home with safety and dignity* [Social Worker] [Canada] [87].

A number of studies highlighted the difficulties which may be encountered by medical practitioners in this regard. In particular, their training and professional experience, which perhaps prioritise the “doctor–patient dyad” over collective working, may act as barriers to team working [42, 63, 70, 80, 87, 88]. In the literature from Australia and Canada, for example, it is clear that there were examples of doctors being open to the idea of interdisciplinary working but also examples of where it clashes with their experience as practitioners with overall responsibility for patient care [42, 80, 87]. In the following example the GP, at the beginning, did not entirely trust the Allied Health Practitioners (Dieticians) and all referrals were the responsibility of the GP.

*[With] better understanding of Diabetes Clinic and services*, *I am more confident in educating patients regarding the benefits of these services* [GP] [Australia] [42].

In Australia and the UK there were data from other health professionals which corroborated this, providing examples of working with doctors who were afraid of change or insistent about how things should be managed [42, 70].

*Yes*, *older [GPs] have been in the practice for a long and they are afraid of changes*, *they don’t want to change and I think we all have to change to go forward* [Nurse] [UK] [70].

### Enrolment

Within the review 17 papers referred to enrolment [39, 44–46, 48, 50, 53, 56, 64, 66, 69, 71–73, 79, 81, 82]. Seven of these were from Canada, four from the UK, three from the USA and one each from Spain, France and Republic of South Africa.

Findings show that health professionals from across disciplinary backgrounds think it is right for them to engage in interdisciplinary team working. There was evidence in the studies reviewed of some enrolment from all professional groups in the review sample.

There was some variation in enrolment *within* professional groups; for example, mixed “buy in” was noted in some studies among physicians [44, 46], pharmacists [46, 73] and nurses [82]. Linked to this, there was variation within specific practice settings [44–46, 69, 79, 82] and over time, if team membership changed [69, 81].

On closer analysis of challenges with enrolment, and in keeping with the aforementioned findings about sense-making, physicians were identified as a professional group that did not get involved with interdisciplinary team working as easily or quickly as other professionals [39, 44–46, 50, 55, 64, 71, 81]. They could resist collaboration both by continuing to work independently rather than joining a team and by withdrawing from a team after a period of time. Their absence from a team disrupted team working significantly [44, 45, 50, 53, 71]. For example, limited physician involvement in team working would mean that there is not enough support for a nurse to fulfil an advanced practice role [72]. In one setting, the original, older physicians of the practice who were resistant to the introduction of a culture of team working were regarded as old fashioned and were encouraged by a new Medical Director to move on in parallel with intentional recruiting of “younger and forward thinking physicians” [44], p. 49].

Conversely, when physicians did get involved in team working initiatives, particularly senior physicians [44, 46, 53], this was a strong lever for driving the work forward. It enabled team working for all health professionals but also was positive for getting other physicians on board. A physician lead or liaison for occupational therapy in a US Family Health Team was identified by OTs as an effective strategy for enhancing enrolment from other physicians to make referrals to the OTs in the team [53].

There were examples of doctors as *the* local champion for interdisciplinary working in certain settings [e.g. [44, 56], or as a core member of a group of champions comprising, for example, physicians working with a dedicated facilitator for primary care team work development [44], GPs with a nurse and practice manager [48] and a project manager and group of doctors [66].

Overall, it was clear that local champions were central to driving interdisciplinary team working forward. With their enthusiasm and vision for interdisciplinary team working, they were identified as key actors for change [44, 45, 48].

Policies for health care reform were identified as an important contextual factor for understanding why professionals got involved in Canada [45], the US [71, 72, 82] and the UK [81]. For example, the 2000 Quebec Government developed a Commission for the Study of Health and Social Services and recommended the implementation of Family Medicine Groups, and the UK Department of Health recommended development of family physician and nurse practitioner collaboration (UK DoH 2003).

Some initiatives for promoting team working came from professional bodies such as the Royal College of General Practitioners in the UK [39], the Canadian Nurses Association and the National Demonstration Project of the American Academy of Family Physicians [44].

Finally, a number of other factors were identified that informed health professionals’ decisions to get involved, including previous positive experiences of team working or current good, trusting relationships with other professionals [73, 82]. The possibility of financial benefits was a driver for some [39, 45, 46, 48, 64]. Having an interest in a specific condition, such as Chronic Kidney Disease that was going to be part of the team work was another relevant factor [48], as well as the general belief in the potential benefits for patients (which resonates with findings described under “Sense-Making”).

*I live*, *breathe and sleep CKD*. *My husband is sick of hearing about CKD and it’s all I talk about*, *so I can’t really be any more committed or interested than I am* [Nurse] [United Kingdom] [48].

*Over the last 20 years I have seen the deterioration in primary care services delivery towards sole practices*. *And each one is always convinced that he is offering the best possible service […] A group practice offers a better service to our patients* [Physician] [Canada][45].

### Enactment

Within the review all 49 papers referred to enactment [39–77], [78, 79], [80–87]. Seventeen were from Canada, 12 from the US, seven from the UK, four from Australia, two each from Sweden and New Zealand, one each from France, Brazil, The Netherlands, Republic of South Africa and Spain.

Focusing on resources for enacting interdisciplinary team work in practice, it was clear that financial resources are extremely significant. The amount of resources available determines team composition, training opportunities [87], information systems for communication between professionals about administrative and clinical issues [44, 45, 53, 62, 64] and the physical spaces available for interdisciplinary team working. Heavy workloads arising from interdisciplinary team working can stretch available resources, if they have not been appropriately increased for team working, and can diminish motivation and participation in team work over time [39, 53].

Remuneration systems in primary care are relevant. Public and private funding models cause tensions in particular, for example in Spain [46] and Canada [79] between pharmacists who are self-employed and GPs who are contracted by the national health services. GPs in Spain are encouraged by the National Health Service to prescribe cheaper medicines and less medicine, while pharmacists have a greater interest in non-rationalisation of medication.

*There are many doctors that say “I’m not giving [prescribing]*, *do you know why*? *Because I’ll get in trouble*, *because they’ll penalise me*.*” They [community pharmacists] think “here it is my money that’s at stake*, *because I have a business and the doctor is a state employed and nothing is going to happen to him/her and he/she doesn’t care … And they must compare this difference of their feeling of responsibility that they a have a business and they must pay a salary to their assistant*, *that there are things to pay for*. *They have an element of entrepreneur that we don’t have* [GP] [Spain] [46].

Another example is regarding GPs in Australia who have concerns about collaborating with Nurse Practitioners with a prescribing role because this will mean a reduction in GP income.

*With the extended primary health care and incentives that GPs have got in their practices…*.*there is quite a significant financial remuneration for GPs…all the doctors see me in terms of pinching the medicare stuff [and) that I am pinching their patients* [Nurse] [Australia] [75].

This highlights interconnections between funding, divisions of labour and trust in each other’s work in the interprofessional network.

Focusing on skills sets, it was clear that training to work as a team is very important to develop appropriate skills. There were examples of this happening by “trial and error” [62] rather than through formal educational fora. A related issue, across countries, is the importance of clarity within the team about each other’s roles and responsibilities. If achieved, this is excellent for team work [48, 70, 83, 86] and it is important for patients as well [60]. Conversely, if it is not achieved, this is associated with tensions in the interprofessional network [60, 62, 63, 73, 75, 82, 86]. For example, physicians do not feel understood by pharmacists and are frustrated by that [73], overlapping roles between nurses and chiropodists are problematic and there is frustration among social workers because their roles are not understood or fully recognised by other primary care professionals [62, 76]. As mentioned above, there are particular tensions among GPs about nurses having prescribing roles that impinge on both a traditional GP role and GP income [75, 87].

Protocols for team working can help to define roles [85], as do interventions in the professional network [44, 53, 66, 69, 75, 76, 85, 87]. Some successful examples identified are nurse practitioners spending time with others in the team to understand their roles, educational backgrounds, information leaflets and meetings to clarify the role of occupational therapists [53], facilitated spaces for team reflection regarding roles [69], information sessions about new nursing roles [45], co-operative inquiry groups [69] and team building meetings [66].

Indeed, the very experience of working together, over time, also enhanced clarity about roles. For example in a Canadian study about collaborative relationships between family physicians and Anticipatory and Preventative Team Care (APTCare) team members, the authors noted that, despite having been formally presented with the role and scope of APTCare colleagues at initiation of the study, it was only through direct interaction in the context of client care that physicians were able to appreciate clearly the roles, scope of practice and individual strengths of the APTCare team members, [80]. Similar findings are evident in these quotes.

*The more contact with the referring doctor*, *the more they [GPs] realise that AHPs play an integral role in the management of their patients in a positive way* [Allied Health Professional (not specified)] [Australia] [42].

*Once people know my role they do check in with me*. *Especially the nurses are much more helpful when it comes to calling back and making sure that the social worker knows because then they know I will follow up*, *which is really good*. *Once I have connection*, *it works out for the good* [Social Worker] [USA] [76].

There were multiple examples of effective and regular interdisciplinary communication about patients and their care in daily practice [39, 61, 68, 69, 73, 74]. Verbal, face-to-face communication was highly valued [66, 85] but communication was often aided (depending on the available resources) by IT systems and the use of Electronic Medical Records as well as electronic patient booking systems [39, 44, 82].

Interactions between team members were often formal, e.g. regular multidisciplinary meetings [39, 53]. Some meetings were during lunchtime or after consultation hours, again depending on the availability of resources for team working [60]. There were also examples of informal and *ad hoc* interactions that were generally described as being positive and effective for shared decision making and informational continuity of care for patients [53, 60, 68, 73]. The value of having co-located teams for formal and informal communication encounters was emphasised in several studies [39, 53, 63, 64, 73, 82, 84, 85, 87].

Overall, it was clear that interactions based on respectful listening and acknowledgment of all professionals’ contributions and expertise were highly valued and most effective [40, 61, 69, 70, 83]. Having fun together was also valued [85]. Thus, respectful interdisciplinary contact emerges as an important lever for developing role clarity and progressing shared patient care in primary care teams [84].

Finally, there were specific findings about skills, roles, confidence and trust in the interdisciplinary network that relate to the role of physicians. Doctors were found to operate with a focus on medical rather than primary care [44, 63], and while other professionals report benefits of sharing patient information and decision making and responsibility [44, 69], this feels risky or uncomfortable *in practice* (as well as at the level of ideation, described under “Sense Making”) for physicians [42, 50].

Some factors that influence the development of divisions of labour in teams appear to be “physician-centric”: roles and responsibilities were decided by physicians’ interests rather than clients’ needs and motivations on the part of other team members to save physicians’ time [63].

Where problems did arise with physicians’ involvement in team working, it was evident that other professionals worked hard to address the issues. For example, pharmacists took steps to gain doctors’ trust rather than vice versa [86], there were expectation of nurses to take first steps to resolve problems with physicians [45], and when doctors didn’t like nurses prescribing, the nurses worked around this by being discreet: a strategy used to continue prescribing without causing too much concern among doctors [75]. Overall, these findings resonate with those presented under “Enrolment”: health professionals from a variety of disciplines work hard to manage interactional difficulties with doctors because it is considered so important to keep physicians on board–without them teams can “fall apart” [73, 75].

There were examples of traditional hierarchies in health care between physicians and other health care professionals across countries impacting on primary care team working [40, 42, 45, 52–56, 68, 70, 74]. One group of authors noted that study participants were not comfortable vocalising their views on this [70].

This hierarchical structure was acknowledged by physicians and described by other professionals. For example:

*I am sure that there are a lot of physicians that do not like the ball being taken from them* [Physician] [Canada] [53].

*I still think there is a hierarchical model*. *They’re never rude [MDs] but there’s an attitude you pick up that you can tell*, *you know* [Nurse practitioner] [USA] [77].

*There is this hierarchy … the GP is at the top and “I’m only a district nurse”*, *the way you are spoken to* [Team Facilitator] [United Kingdom] [68].

### Appraisal

Only 10 papers made reference to appraisal [39, 42, 44, 47, 56, 58, 66, 71, 81, 85]. Two of these were from Canada, five from the USA, two from the UK and one from Australia.

Of the papers related to appraisal, six clearly reported the use of formal evaluations with health care professionals [39, 44, 47, 56, 58, 85]. The models or frameworks used for formal evaluation were LEAN [85], Reflective Adaptive Process [47, 56], a National Demonstration Project [44], a workshop to enhance interdisciplinary team work [58] and a Quality Team Development initiative [39]. Interestingly, the process of formal evaluation was in fact helpful for enabling and supporting team working and development [39, 44, 47, 56, 58, 85]. Following a Reflective Adaptive Process it was noted that:

*Meeting once a week has made our practice run so much smoother*. *We were having problems a year ago between the offices*, *but they’ve almost disappeared now*. *We make sure that new people always come to the meetings right away*. *They make people better at team work*. *This fosters collaboration*. *We use it to get a lot accomplished* [Physician] [USA] [56].

Two papers clearly mentioned reliance on informal feedback between health care professionals about their interdisciplinary work together [66, 81]. Furthermore, there were two examples of patient evaluations [42, 71]. Patients found meetings with their pharmacist an “incentive” to adhere to their medication [Patient] [Canada] [71]. In an Australian paper patients provided feedback to their GPs regarding their consultations with Allied Health Professionals. Three-way communications took place by phone between the GP, patient and AHP to track progress and to negotiate goals [42]. Patients found meetings to be beneficial. In addition they acknowledged the expertise offered by the different interdisciplinary team members. As one patient very eloquently put it:

*No one person has everything in their roughly two kilo of fat and water inside their cranium*. *Therefore*, *getting more than one dollop of cortex working on my problem […] may in fact be to my great benefit* [Patient] [Canada] [71].

Overall, the findings of all types of evaluations were broadly positive about the team working process and patient care, within and across professional groups. The informal feedback on the team referred to high satisfaction among the participants whereby they would like the partnerships to continue and expand [44, 66]].

*I would say we are on the road […] it’s just a really long journey*. *At this point I’d say we’ve got a map and we are driving on the right route* [Doctor] [USA] [44].

In terms of identifying issues that would support and sustain team working, most studies highlighted the value of introducing financial incentives, the need to improve communication with regular interdisciplinary meetings, enhanced opportunities for shared decision making between professionals, improving the mutual understanding of team members’ roles and improving the teams’ shared goals and vision.

These ideas for reconfiguration strongly resonate with the identified barriers under sense-making, enrolment and enactment.

## Discussion

To the best of our knowledge, this is the first integrative review to use a theoretical framework to examine primary care professionals’ accounts of interdisciplinary team working in primary care. This analysis provides a novel contribution to the literature because it maps these accounts onto Normalisation Process Theory, thus providing a comprehensive conceptual analysis of facilitators and barriers to implementation. The analysis also highlights gaps in the literature from which to highlight directions for future research.

A thorough and systematic search of reviews published between 2004 and 2014 identified 49 papers on interdisciplinary team working in primary care. Eleven countries were represented and most papers were from Canada (*n* = 17). Following SIGN, the majority of papers represent level 3, qualitative case studies. This study design is appropriate to the review question, which focuses on team working in practice rather than on interventions or evaluations of impact. The overall quality of the qualitative papers reviewed is good. The spread of publications over time is 10 years, with a steady increase in papers from 2010 to date, reflecting the emphasis on primary care within international policy.

The majority of papers relate to experiences of family physicians, nurses and pharmacists, with fewer papers relating to the wider network of health professionals. This is problematic because primary care is reliant on a wide network of health professionals who have a shared focus on patient care but differential knowledge and skills to bring to bear on the work. The literature needs to reflect all their professional views and experiences.

### Summary of findings

The papers in this review have been analysed and presented under the four constructs of NPT. For *Sense Making*, the key finding in the available literature (*n* = 17) was that the idea of interdisciplinary team working in primary care usually makes sense to service providers as they have a shared view that it will have potential value and gains for patient care. However, the potential value of sharing care and responsibility of patients with other health professionals is not necessarily clear to some doctors, particularly older ones.

For enrolment (*n* = 17) there is evidence of both “buy in” and resistance from primary care professionals, across country settings and within different local practice settings. Champions, with vision and drive to galvanise the network and co-ordinate team working, were a key facilitator. Physicians’ involvement was crucial in this regard as they were identified as particularly effective champions but, also, the most resistant professional group. If they do not engage it can limit the scope for interdisciplinary work between other health professionals.

All of the papers in this review had material that related to *Enactment*. There was a dearth of explicit or detailed analysis about the policy and governance models that shape the implementation process. However, it does seem that mixed funding models are problematic because they can undermine the trust health professionals have in each other’s roles (protecting professional territory) or motivations for decisions about patient care (the best treatment versus one with a commercial benefit). There were also relatively few data about skills for team working, although a pattern of “learning by doing” was evident. Clarity and trust about divisions of labour, respect for each other’s roles, and respectful and regular communication (preferably face to face, although e-communication has value too) are frequently reported as facilitators for smooth team working. There are specific findings about traditional hierarchies between medicine and other professions that shape the relational and interactional dynamics mentioned above.

There were only 10 papers that related to the fourth construct, *Appraisal*. These highlighted that there is lack of appraisal or auditing of team working. The available evaluations, both formal and informal, indicate high satisfaction with interdisciplinary team work. Furthermore, evaluation processes can support the development of team work, as problems were identified, explored and, sometimes, resolved.

### Connections with literature

This review confirms that the implementation of top-down policies for interdisciplinary working is only partially successful [11, 12]: this is not a routine way of practice in primary care in many health care jurisdictions studied. Even where it is a more usual feature, such as in the UK, the US and Sweden, there are challenges in practice.

The available literature suggests that this way of working makes sense to many health professionals and is regarded as a positive approach for improving patient care and outcomes. These findings resonate with policy drivers for interdisciplinary team working in primary care [4–8, 89].

There are several descriptions of inter-related enrolment and enactment problems with physicians in primary care teams. These problems are connected with socialisation processes and traditional hierarchies in health care. Doctors are trained to manage patient cases individually as opposed to collectively, having final/sole responsibility for patients and authority over other professional colleagues [20, 23, 89, 90]. However, no studies were identified that explored how legitimate physicians thought it was for them to be involved in team working. There was no research focused on methods for increasing enrolment of primary care physicians, or indeed other professionals.

In keeping with the literature [15, 16, 91], there is a lot of evidence that the nature and regularity of communication between primary care professionals is a key factor in team working. Where communication is frequent and respectful and where there is clarity about roles and divisions of labour, team working is successful. Indeed, it appears that frequent, respectful communication can also be a lever to reducing role confusion, overlapping roles, and poor trust in each other’s work. Such communication may be a function of structures for formal clinical meetings, dedicated events or initiatives to support teams or formal appraisal process. In keeping with other literature [16, 17, 22], funding models and being co-located seem important, although there is lack of explicit and focused analysis of these very important contextual factors.

Overall, and in keeping with previous literature [15–17, 20], primary care health professionals with experience of successful team working report a range of benefits for patient care which they highly value. These have been identified both formally and informally but, overall, more empirical work is required to expand knowledge about the perceived benefits among service providers. It would be interesting to establish how they might differ between the full ranges of professional groups involved in primary care delivery. This should, of course, be compared with perceived benefits among patients who are in receipt of team care.

To the best of our knowledge this is the first integrative review using a theoretical framework to review international literature about interdisciplinary team working in practice. Whilst there is a dearth of evidence in terms of the experiences of all primary care professionals, a key lever for interdisciplinary team working is to get professionals working together and learning from each other in practice.

### Methodological critique

We were rigorous in our approaches to all stages of the review, which adhered to the PRISMA guidelines. Whilst the majority of studies in the review were qualitative, due to the nature of the subject area, a rigorous systematic process was adhered to. A systematic review of RCTs was not possible. At all stages at least two reviewers worked together. We had frequent discussions among the whole team to refine and develop our synthesis and interpretations. Using the NPT framework enabled us to discern key features of the existing evidence base drawn from an international perspective, thus increasing the potential transferability of these findings to a range of contexts. The use of a robust theoretical framework for analysis and synthesis was important given that the evidence (SIGN Level 3) which exists is mainly qualitative.

### Implications for future research

The following section outlines research gaps and questions, which when addressed, should help contribute to a coherent, theoretically informed body of evidence.

First, there is a need for research that explores the experiences of primary care team working among the *full network* of primary care professionals.

Second, based on our use of NPT, it is clear that there is a lack of research about important areas of implementation work, namely sense making, enrolment and appraisal. In relation to *sense making*, further research on the concept of shared vision amongst health care professionals is required. Whilst this review found broad agreement about the value of interdisciplinary team working to improve patient care, how different professional interpret this, based on their training and professional identity, can differ for example on issues such as risk, rights and enablement. Therefore, whilst there may be ‘buy-in’ by all to improve patient care, how each professional group interprets this may have implications for functional interdisciplinary team working. It will also be important to establish what methods are best for promoting a shared vison for team working. Is this something amenable to training or continuing education or is it more an aspect of social mores and culture that is best achieved through mentoring and modelling? Further investigation is required, in terms of establishing methods for promoting *enrolment*, by professional groups, that explicitly attend to professional socialisation and perceptions of legitimacy for participation in interdisciplinary teams. As highlighted in the review, physicians did not get involved with interdisciplinary team working as easily or quickly as other health care professionals, consequently further research is required in identifying why and how professionals, especially doctors, can be best supported to work within teams. In terms of *appraisal* it would be beneficial to evaluate how health care professional appraise team working in practice. It may also be valuable to identify how within-team communication affects both work satisfaction and communication with patients and families.

Third, to add to the existing evidence in this review about *enactment* of team work in practice, there needs to be more empirical analysis about the policy and governance factors that shape team working in daily practice and analysis of the specific impacts of co-location and/or electronic communication on positive communicative encounters between professionals.

Finally, *participatory*, *implementation studies* that investigate and support interdisciplinary team working should be conducted. These could explore problems experienced in practice to identify shared and mutually beneficially solutions (see De Brun et al [14]). These are warranted given the pattern in the literature that reflective team work in practice can, in fact, be a facilitator for team functioning.

## Conclusion

This innovative, NPT-informed systematic review has shown that a key lever for interdisciplinary team working in primary care is to get professionals working together, to know each other and to learn from each other in practice. However, the evidence base is limited at present because it does not reflect the experiences of all primary care professionals (it is dominated by research from doctors and nurses), it relates to a small number of countries, and it is primarily about the enactment of team working. This limits the scope to draw firm, generalisable conclusions about levers and barriers to implementation of interdisciplinary working in primary care. To progress, we need to know much more about the experiences of the full network of primary care professionals and about all aspects of implementation work.

## Supporting information

S1 Checklist PRISMA checklist. (PDF)

S1 Table Summary of studies included in final analysis. (PDF)

S1 Text Review Protocol—International Prospective Register of Systematic Reviews PROSPERO 2015: CRD42015019362. (PDF)
