# Correction: Assessing the facilitators and barriers of interdisciplinary team working in primary care using normalisation process theory: An integrative review

**DOI:** 10.1371/journal.pone.0181893

**Published:** 2017-07-24

**Authors:** Pauline O’Reilly, Siew Hwa Lee, Madeleine O’Sullivan, Walter Cullen, Catriona Kennedy, Anne MacFarlane

There is an error in the first sentence of the second paragraph of the Discussion section. The correct sentence is: A thorough and systematic search of reviews published between 2004 and 2015 identified 49 papers on interdisciplinary team working in primary care.

In Fig 1, there should be an arrow from the box ‘207 full-text articles assessed for eligibility’ to the box ‘158 full-text articles excluded with reasons’. Please see the corrected Fig 1 here.

**Fig 1:**
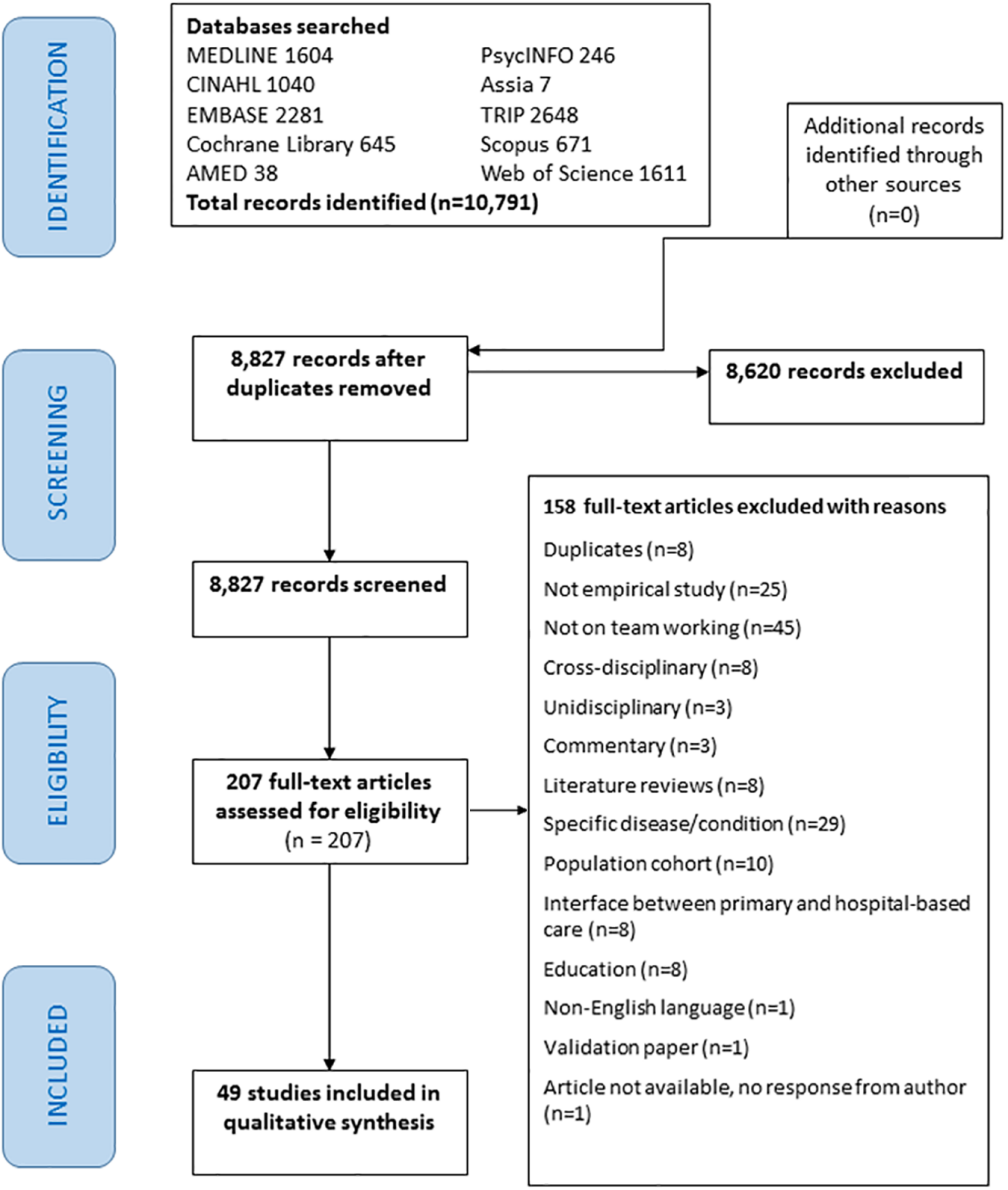
PRISMA flow diagram [31].
