# Supplementary material for: Assessing the facilitators and barriers of interdisciplinary team working in primary care using normalisation process theory: An integrative review
Source: PLoS One. 2017 May 18;12(5):e0177026. doi: 10.1371/journal.pone.0177026 (PMC5436644; doi:10.1371/journal.pone.0177026)
Supplement: S1 Table — (PDF) [file pone.0177026.s002.pdf]

S1 Table: Summary of studies included in final analysis

Ref. no.	Authors	Country	Setting	Study aim	Participants	Study design	Main findings related to interdisciplinary working
39	Macfarlane et al. (2004)	UK	Primary Care organisations (PCOs)	To evaluate Quality Team Development from the perspective of participants and assessors	*N* = 30. 11 GPs, 8 nurses, 2 administrators, 9 managers	Qualitative retrospective interviews	Clear evidence of positive changes in relationships between professionals and interdisciplinary practice because of team working, but queries about sustainability.
40	Nemeth et al. (2008)	USA	Primary care practices within a research network clinical trial	To explore the process of change used to implement clinical guidelines for primary and secondary prevention of cardiovascular disease in primary care	28 staff members: 10 physician clinicians, 1 physician assistant, 1 clinician nurse practitioner, 8 clerical staff, 8 clinical support	Qualitative methods: observation and interviews	Value of involving the team and developing team working to enable implementation of guidelines.
41	Pullon et al. (2009)	New Zealand	Primary care: urban and suburban region	To explore organisational factors that affect the ability of primary healthcare professionals to work in effective teams	*N* = 18 (9 nurses and 9 doctors)	Qualitative retrospective interviews	Issues of resources are key and can undermine interest in and capacity for team working. Good overall business practice as the key to successful teamwork, not the contractual arrangement alone.
42	Chan et al. (2010)	Australia	Multidisciplinary teams within general practices and external collaborations	To explore changes to team work after an intervention to improve collaborations	35 GPs and 39 allied health providers	Qualitative methods: intervention facilitator reports, GP reports, AHP survey	Improved collaboration depended on understanding and trust between professionals and the development of personal relationships among the team members, facilitated by face-to-face or telephone meetings and discussion of patients' needs.
43	Chesluk & Holmboe (2010)	USA	Primary care practices representing variety of practice configurations	To explore how the entire primary care practice team works together in the course of caring for patients	5 physicians, 19 professional and administrative staff and 9 patients	Qualitative ethnography observations and interviews	Current practice systems are not designed to elicit and integrate their full individual and group capabilities.

Ref. no.	Authors	Country	Setting	Study aim	Participants	Study design	Main findings related to interdisciplinary working
44	Nutting et al. (2010)	USA	Practices in 25States: rural, suburban and urban areas.	To explore experience of practices in transitioning toward patient-centred medical homes (PCMHs) in the National Demonstration Project (NDP)	*N* not specified	Qualitative–multimethod evaluation strategy, analysing data from direct observation, depth interviews, e-mail streams, medical records, and patient and practice surveys	PCMH requires more than a sequence of discrete changes. The practice transformation process may be fostered by promoting adaptive reserve and local control of the developmental pathway.
45	Rodriguez & Pozzebon (2010)	Canada	Urban Family Medicine Group	To understand the social construction of the identity of a Family Medicine Group	*N* = 11 key physicians, nurses, and administrative staff (breakdown not provided)	In-depth longitudinal case study using qualitative interviews and observation methods	Professionals need to have shared time/space contexts to communicate and overcome interprofessional and interpersonal conflicts. They need to make sense of the work and define who they collectively are and what they do as a clinical team.
46	Rubio-Valera et al. (2012)	Spain	Two regions with contrasting primary care governance and funding	To analyse barriers and facilitators in collaboration between general practitioners and community pharmacists in Spain	*N* = 37. 18 GPs and 19 community pharmacists	Descriptive–exploratory qualitative study using interviews	Many factors act as barriers to collaboration: professionals' attitudes, experiences and their perceptions of usefulness of working together.
47	Shaw et al. (2012)	USA	25 primary care practices participating in a federally funded QI trial	To identify new understandings of team-based reflection	*N* not specified. 4 primary care practices selected from 15. Team comprised office manager, physician, nurses and medical assistants	Descriptive–exploratory qualitative study using interviews	Many of the social and relational elements that often hinder effective change can be addressed through open and critical reflection by team members.
48	Kislov et al. (2012)	UK	Four general practice settings as individual subcases – two small and two large practices	To explore the effects of intra-organisational and inter-organisational boundaries on the implementation of service improvement within and across primary healthcare settings and on the development of multiprofessional and multi-	*N* = 12: 5 doctors, 3 nurses and 4 practice managers	Qualitative embedded case study design	It is important to map existing CoP landscapes in primary care that are relevant for a given service improvement initiative, analysing the configuration of boundaries, roles and identities in these landscapes, and combining external and internal facilitation to make the boundaries between all CoPs involved more permeable and to enable the incremental development of these CoPs through participation in the initiative.

Ref. no.	Authors	Country	Setting	Study aim	Participants	Study design	Main findings related to interdisciplinary working
				organisational communities of practices (CoPs) during this process			
49	Howard et al. (2012)	USA	60 primary care practices randomised into intervention and control groups	To identify the strategies used in inclusive leadership	*N* not specified	Qualitative descriptive study	It is not enough to intellectualise leadership inclusiveness: it must be made real (listen and involve people and be respectful) for more effective collaboration within teams.
50	Vedel et al. (2013)	France	Primary care in a French setting	To identify and obtain a better understanding of CTM adoption factors and the diffusion process	*N* = 55: 40 primary care physicians, 15 nurses	Qualitative longitudinal case study	CTM diffusion is a social phenomenon that requires a major commitment by clinicians and a willingness to take risks; the role of opinion leaders is key. Paying attention to the notion of a critical mass of adopters is essential to develop implementation strategies that will accelerate the adoption process by clinicians.
51	Vachon et al. (2013)	Canada	Primary care setting in Quebec's Monterefie region	To describe the theory underlying an interprofessional educational intervention developed for the purpose of improving chronic disease management in primary care	*N* not specified. Convenience sample of physicians, nurses and pharmacists	Qualitative, using a programme impact theory-driven approach	The 3-hour education intervention improved interprofessional collaboration and quality of care. However, a 3-hour workshop alone cannot lead to major changes in practice. Long-term interventions are needed to support this complex change process.
52	MacNaught on et al (2013)	Canada	Primary care setting	To understand how roles are constructed within interprofessional health care teams in primary care	*N* not specified. Two case studies comprised nurses, pharmacists, social workers and dieticians	Qualitative comparative case study approach	The findings indicate that role boundaries can be organised around interprofessional interactions (giving rise to autonomous or collaborative roles) as well as the distribution of tasks (giving rise to interchangeable or differentiated roles). There are structural, interpersonal and individual influences on role construction.

Ref. no.	Authors	Country	Setting	Study aim	Participants	Study design	Main findings related to interdisciplinary working
53	Donnelly et al. (2013)	Canada	Primary health care setting, Ontario	To explore what structures and processes support the integration of occupational therapy in Family Health Teams	Purposeful sample of 4 sites which included chiropodists, psychologists, social workers, dieticians, physician assistants, pharmacists, patient educators, mental health workers, health promoters, respiratory therapists, case managers and nurses	Qualitative case study	Each Family Health Team had a unique organisational structure that contributed to the integration of occupational therapy. Communication, trust and understanding of occupational therapy were key elements in the integration of occupational therapy into Family Health Teams, and were supported by a number of strategies including co-location, electronic medical records and team meetings.
54	Altersved et al. (2011)	Sweden	Primary health care setting	To describe health-care teams' experiences of the new Advanced Practice Nurse role and investigate what opportunities and barriers to the role exist	*N* = 81, GPs, nurses, biomedical analysts and secretaries	Qualitative descriptive study	There are many advantages to the work flow within a PCT in having APNs involved. There were some disadvantages including extra work for other members of team.
55	Bailey et al. (2006)	Canada	4 rural Ontario primary care practices	To understand the experience of the FPs and NPs of working in collaborative practice	*N* =18: 13 physicians and 5 nurses	Qualitative descriptive study	For collaborative practice it is important to clarify the scope of shared practice and to enhance trust and address ideological differences regarding disease prevention and health promotion. Collaborative relationships evolve over time.
56	Balasubramanian et al. (2010)	USA	Primary care setting	To examine how practices in a National Demonstration Project engaged in a team-based collaborative change management strategy and the types of issues they addressed	*N* not specified;.25 intervention practices	Qualitative descriptive study.	Transitioning to a new paradigm of primary care is far more complex than merely implementing a series of changes. Neither incremental quality improvement nor learning organisation facilitation alone will work.

Ref. no.	Authors	Country	Setting	Study aim	Participants	Study design	Main findings related to interdisciplinary working
57	Boon et al (2009)	Canada	Primary care teams in Ontario	To explore what the terms 'integration' and 'collaboration' mean for practitioners and other key informants working in multiprofessional health care teams	*N* = 16, comprising chiropractors (5), physicians (4), midwife (1), nurse practitioners (2), physio (1), other admin or academic roles (3)	Qualitative grounded theory	Most participants differentiated collaboration from integration. They generally described a model of professions working closely together (i.e. collaborating) in the delivery of care but not subsumed into a single organisational framework (i.e. integration).
58	Cashman et al. (2004)	USA	Federally funded community health centre in New England, USA.	To report findings from a longitudinal study of an intervention to enhance interdisciplinary team functioning in a primary care setting	*N* not specified. Health centre comprised physician, nurse practitioner, physician assistant and health assistant	Qualitative longitudinal study	(a) The heterogeneity of team composition, (b) role conflict and role overload, (c) constraints placed on members by the larger organisational structure, and (d) members' lack of knowledge about the process of team development were identified as important factors influencing team working.
59	Davey et al. (2005)	UK	Two London boroughs, one NHS Community Health Services Trust, four Primary Care Groups and two social services departments.	To investigate perceptions of joint working in social services and general practice	*N* = 66, 52 social workers and 14 GPs	Qualitative descriptive study.	Lack of awareness of each other's role prevails. While GPs and social workers agree that joint working should have advantages for their shared clients/patients, each profession wants the other to change its way of working.
60	Elissen et al. (2011)	The Netherlan ds	Primary care setting in Limburg	To explore explanation for the difficulties associated with the emergence of multidisciplinary co-operation in primary care	*N* not specified. GPs, obstetricians, physical therapists, exercise therapists, speech therapists and psychologists	Qualitative design using interviews and document analysis	Respondents' current co-operative actions fall short of not only their own ambitions, but also the true essence of multidisciplinary co-operation, which reaches further than the often reported ad hoc consultations in hallways and during coffee breaks.

Ref. no.	Authors	Country	Setting	Study aim	Participants	Study design	Main findings related to interdisciplinary working
61	Fernandes Biffe Peres et al. (2011)	Brazil	Primary health care teams in São Paulo	To understand the inclusion of the ACS in the working process of primary health care teams by analysing their strengths and weaknesses, from their own perspective	*N* not specified. Convenience sample	Qualitative descriptive study	ACSs reveal that the easy aspects of working as a team depend on the construction of interpersonal relationships, including the possibility of having discussions about everyday problems, liberty to speak, communication and dialogue, attitudes of respect, common language, willing to learn, co-responsibility, and bonding.
62	Fouche et al. (2013)	New Zealand	Primary and community health care setting	To explore the potential for collaboration between social work and community pharmacy in the context of primary and community health care	Phase 1 *N* = 21, Phase 2 *N* = 32, physical therapy , speech and language therapists, nurses, pharmacists, social workers, occupational therapists and GPs	Two-phase qualitative study	The main benefits of interprofessional collaboration between these health professionals were reported as the pooling of skills and knowledge, and medicines management. Strategies offered to facilitate an increasingly effective working relationship between social workers and community pharmacists mostly focused on interprofessional education, which would enable an increased (and timely) understanding of each other’s roles as well.
63	Hills et al. (2007)	Canada	Community setting within a city in Canada	To explore the challenges of putting the multidisciplinary practice approach into practice	*N* not specified. Primary care practitioners and community members who had received health services: no specifics given	Qualitative, using a modified critical incident technique and a Freirean approach	The successful implementation of an MDP approach to PHC requires a movement away from physician-driven care. This can only be accomplished after changing the underlying structures, values, power relations, and definition of roles within the health care system and the community at large.
64	Knowles et al. (2013)	UK	Naturalistic National Health setting	To explore the extent to which collaborative care was implemented in a naturalistic National Health Service setting	*N* = 23. Psychological well-being practitioners and nurses	Qualitative longitudinal exploratory study	Implementation of collaborative care in routine settings can be hindered by (1) lack of engagement with the organisational aspects of the model, which may be neglected even if the patient level factors of continuity of care and holistic care are valued; (2) pre-existing structures and norms that emphasise division of labour between the provision of physical and mental health care.

Ref. no.	Authors	Country	Setting	Study aim	Participants	Study design	Main findings related to interdisciplinary working
65	Kolodziejak et al. (2010)	Canada	Primary health care setting	To provide guidance on how to integrate a pharmacist into an already established primary healthcare team	*N* not specified. Physicians, psychiatrists, nurses, dietician, social worker, massage therapist, chiropractor, health educator, internal medicine/obstetrics/gynaecology	Qualitative action research	Eight steps to support the effective integration of a pharmacist into a PCT are provided and include the need for attention to role clarification for pharmacists, credibility for pharmacists within the team, specifying logistics for pharmacists to have direct patient care, conduct patient consultations and refining integration based on the experience and patient feedback.
66	Korabek et al. (2004)	Canada	Primary care setting	To describe the Canadian experience related to the development, implementation, and evaluation of the primary care relationship between community-based physicians and nurses	*N* = 4221 nurses, 21 physicians	Qualitative study	Following the principles of a collaborative partnership, continuance of a formal orientation process involving the orientation of one partnering group at a time is recommended. Also, each partnership is unique, and the more choice given to the participants as to whether they form a partnership and with whom they partner, the greater the satisfaction and commitment to working collaboratively.
67	Kvarnstrom et al. (2006)	Sweden	Multi-professional healthcare team in south east Sweden	To explore how members of multi-professional healthcare teams talk about their team	*N* = 32: nurses, physiotherapists, physicians, occupational therapists, medical social workers, speech therapists, administrators and psychologist	Qualitative using discourse analysis	Discursive patterns provided powerful rhetorical resources for team members both to affirm membership and to claim superiority.
68	Mahmood-Yousuf et al. (2008)	UK	Primary care setting in England	To investigate the extent to which the Gold Standard framework influences interprofessional relationships and communication, and to compare GPs’ and nurses’ experiences	*N* = 3817 GPs, 19 nurses and 2 framework facilitators	Qualitative case studies	Implementing the framework enabled processes of communication associated with high-quality palliative care in general practice, but there was marked variation in how this worked in individual teams. In general, hierarchical doctor–nurse relationships persisted.

Ref. no.	Authors	Country	Setting	Study aim	Participants	Study design	Main findings related to interdisciplinary working
69	Mash et al. (2008)	Republic of South Africa	Community Health Centre in Cape Winelands District of the Western Cape	To explore experience of both unsuccessful and successful attempts to introduce practice teams of doctors and nurses	*N* not specified	Qualitative – an emergent action research study design	The ability to self-organise and create new structures; the importance of participation in planning, monitoring and evaluation; and organisational resilience which values local autonomy while creating a collective vision, identity and values are attributes of the successful creation of practice teams.
70	Shaw et al. (2005)	UK	Primary care setting in an inner city area of southeast London.	To explore whether primary healthcare professionals in these practices felt that progress with Personal Medical Services was underpinned by effective teamworking.	*N*=48 from 21 practices comprising GPs, nurses and practice managers	Qualitative	Team working is limited by a lack of shared objectives, problems with recruitment, poor communication and hierarchical structures.
71	Shaw et al. (2008)	Canada	Urban family health centre	To explore the complexities of patients' experiences of interprofessional care	*N* = 10; purposive sampling; 3 healthcare professionals (family physician, social worker and pharmacist); 7 patients, all English speaking (aged 25–88 years)	Qualitative study using narrative analysis	Interviews with health professionals revealed that while the delivery of care was rooted in a patient-centred model, professionals, at points, struggled to find common ground with patients and initiated interprofessional care as a strategy for grappling with this conflict.
72	Solheim et al. (2007)	USA	Primary health care setting	To better understand team-based Primary Health Care practice in the US	Faculty/staff in a Midwestern nursing college (*N* = 94) and interviewing a purposive sample of nursing faculty/staff identified with PHC (*n* = 10) and their health professional collaborators (*n* = 10).	Qualitative study	Team working is effective if there is a common goal or mission. Leadership is important to achieve this mission. Setting ground rules, expectations, resources, establishing communication and a democratic team process will enhance balanced team dynamics.

Ref. no.	Authors	Country	Setting	Study aim	Participants	Study design	Main findings related to interdisciplinary working
73	Tan et al. (2014)	Australia	Primary care setting	To elicit the views of Australian GPs and pharmacists on the integration of pharmacists into the general practice setting, the proposed roles for a general practice pharmacist, and the factors influencing integration	*N* = 2711 GPs and 16 pharmacists	Qualitative descriptive study	The potential roles for practice pharmacists were deemed to be multifaceted and in some cases allowed for role expansion. These roles were thought to offer potential benefits and disadvantages. The integration of pharmacists into general practice was believed to be hindered by limited funding and infrastructure and by practitioner perceptions.
74	Van et al. (2011)	Australia	Metropolitan and rural New South Wales, Australia	To investigate (1) the nature and extent of interactions between GPs and community pharmacists; (2) the factors that influence these interactions in the context of professional pharmacy services	*N* = 23 GP–pharmacist pairs	Qualitative study using a grounded theory approach	Provision of a service can be a trigger for team working. Factors that influence collaborative behaviours include interactional, practitioner and environmental determinants. Facilitators of collaboration in primary care are presence of rules and protocols, inter-professional continuing education and availability of remunerations.
75	Wilson et al. (2005)	Australia	Multi-site study: urban, rural and remote clinics and hospitals throughout New South Wales	To explore contemporary collaborative experiences of nurse practitioners (NPs) in providing care with general practitioners (GPs) and allied health care professionals	*N* = 9 nurses	Qualitative descriptive exploratory design	Most NPs reported dissatisfaction from working in ineffective collaborative relationships with medical and allied health care professionals. Total collaboration did not automatically occur and was identified as the exception. Health care providers need to acknowledge each other’s unique, valuable contribution.
76	Young et al. (2009)	USA	Community-based settings in a medium-sized city in the mid-eastern section of the United States	To elicit how nurse and social work case managers conceptualise and practice advocacy and how their professional relationships facilitate this process	*N* = 2015 nurses and 5 social workers	Qualitative research using interviews and field notes.	Trust and communication are important to facilitate advocacy, as is the setting, others’ perceptions of case managers’ knowledge and expertise, and power dynamics between professionals. The ability of case managers to effectively advocate is greatly influenced by the strength of the relationships they forge.
77	Martin et al. (2008)	USA	Collaborative health care team in large suburban area	To analyse contradictions occurring between medical doctors and nurse practitioners working collaboratively on a health care team	*N* = 13 (5 physicians and 8 Advanced Practice Nurses)	Qualitative retrospective interviews	Identification of contradictions in communication in physician–APN interactions that can provide insight into how the quality of collaboration might be improved.

Ref. no.	Authors	Country	Setting	Study aim	Participants	Study design	Main findings related to interdisciplinary working
78	Dieleman et al. (2004)	Canada	Community-based teams providing care to high-risk community dwelling individuals	To examine the perceptions of pharmacists, physicians and nurses as they worked together in community-based teams to provide care to 199 high-risk community dwelling individuals	*N* = 22	Quantitative study	The providers found that working in a team environment was very useful when they dealt with complex primary-care patients. They identified open communication, respect for other team members, understanding of their roles and expertise, and being open to learning as important for collaboration.
79	Drew et al. (2010)	Canada	Primary care setting in Alberta	To explore the level of perceived team effectiveness in Primary Care Networks (PCNs). A secondary exploratory objective was to identify strategies, including team composition, that relate to team effectiveness in the PCNs	*N* = 115 physicians, nurses and administrators	Quantitative: descriptive, cross-sectional, exploratory	There is a need for strategies to focus on regular and frequent meetings as a communication tool in the primary care team setting. Areas of relative weakness are team partnership, team purpose and vision and team roles. A redistribution of resources (time, money, energy) to these areas might help teams become better rounded.
80	Legault et al. (2012)	Canada	Established rural family practice	To describe the development of a collaborative relationship among primary care professionals with different remuneration models	*N* not specified	Mixed methods: qualitative interviews and focus groups study, analysis of family practice staff log books, quantitative survey with FPs, NPs, and the pharmacist	Regardless of patient population and sector of health care, developing collaborative relationships and learning to work collaboratively is difficult and takes time. Teams need ongoing support and education about how to make collaborative care practices work
81	Bradley et al. (2008)	UK	Primary care setting in England	To investigate interprofessional collaboration between general practitioners and pharmacists involved in the delivery of enhanced pharmacy services under the local pharmaceutical services (LPS) contract in England	Stage 1 questionnaire (*n* = 31 at stage 1 and *n* = 27 at stage 2) and in-depth case study of six of the 13 operational Local Pharmaceutical Services (LPS) contract sites. Semi-structured interviews with pharmacists/providers (*n* = 20), GPs (*n* = 13) and PCT Trust representatives (*n* =	Mixed-methods study	GPs seem to have most control over shaping and controlling how the team functions. Co-location does not guarantee cooperation: building trusting relationships takes time.

Ref. no.	Authors	Country	Setting	Study aim	Participants	Study design	Main findings related to interdisciplinary working
					6).		
82	Drummond et al. (2012)	Canada	General, community-based, primary health care clinical settings	To explore the status and processes of interprofessional work environments and the implications for interprofessional education in a sample of family medicine teaching clinics	*N* = 307 FPs, 9 RNs, 5 Licensed PNs, 2 residents, 1 psychologist, 1 informatics, 1 pharmacist, 1 dietician,1 nurse, 1 receptionist, 1 respiratory therapist	Mixed-methods iterative study.	The presence or absence of leadership focused on interprofessional collaborative clinical work appears to be fundamental to the development and sustainability both of interprofessional practices themselves and of the interprofessional education associated with them.
83	Garner et al. (2008)	Canada	Two Community Health Centres in Ottawa, Ontario	To investigate the effect of integrating chiropractice on the attitudes of providers on two healthcare teams	*N* not specified:.physicians, nurse practitioners and degree trained nurses.	Mixed-methods case study design	The project demonstrated the importance of structuring and scheduling interactions between the established health care team and the chiropractors to facilitate a successful integration. This integration had a positive impact on providers' individual practices and changed opinions and views of healthcare practitioners toward chiropractic, resulting in conventional and nonconventional healthcare providers working together on a collaborative healthcare team
84	Humbert et al. (2007)	Canada	A Family Health Network in rural Ontario	To evaluate whether integrating NPs and a pharmacist was an effective approach for the management of patients living with multiple chronic illnesses	RCT: patients *n* = 120 in the intervention arm and *n* = 121 in the control group; *n* = 8 physicians, *n* = 3 half-time NPs for the duration of the 18 month study, *n* = 1 full time pharmacist for 15 month period	Mixed-methods study including a random control trial	Integration of the NP role into a family practice requires role clarification, an understanding of scopes of practice, excellent communication, co-ordination of care, individual respect and trust, and willingness to change the ways of providing primary health care, focusing on patients with multiple chronic illnesses in the home setting. A shared-care collaborative approach improved the quality of care delivered to these patients.
85	Sinsky et al. (2013)	USA	Different geographic regions..Small private practices, large integrated delivery systems,	To explore whether there are places where physicians and other staff are thriving and whether some practices have found innovative solutions to the challenges of office organisation	*N*=200Physicians	Mixed-methods study	A shift from a physician-centric model of work distribution and responsibility to a shared-care model with a higher level of clinical support staff per physician and frequent forums for communication can result in high-functioning teams, improved satisfaction, and greater joy in practice.

Ref. no.	Authors	Country	Setting	Study aim	Participants	Study design	Main findings related to interdisciplinary working
			academic medical centres				
86	Snyder et al. (2010)	USA	A relatively small community setting	To describe the professional exchanges that occurred between community pharmacists and physicians engaged in successful CWRs (collaborative working relationships)	*N* = 178 physicians and pharmacists.	Mixed-methods study	Pharmacists are usually the ones who initiate relationships with their physicians. Physicians see themselves as responsible in the team and therefore consider themselves decision makers.
87	Wilson et al. (2005)	Canada	Capital Health region, Edmonton, Alberta	To determine how FPs/GPs view the issues associated with increased involvement in collaborative practice and their current and potential future working relationships with other health professions	*N* = 583: survey of family physicians and GPs. 9 focus groups conducted with 46 FPs/GPs.	Mixed-methods study	Interdisciplinary collaborative practice is influenced by quality and capacity of care, quality of work life, affordability, availability/accessibility of other health professionals, team-building processes, responsibility/accountability, and system resources. Interest in interdisciplinary working is much greater than actual experience of it in practice.
