# Supplementary material for: Assessing the facilitators and barriers of interdisciplinary team working in primary care using normalisation process theory: An integrative review
Source: PLoS One. 2017 May 18;12(5):e0177026. doi: 10.1371/journal.pone.0177026 (PMC5436644; doi:10.1371/journal.pone.0177026)
Supplement: S1 Text — (PDF) [file pone.0177026.s003.pdf]

Assessing the facilitators and barriers of interdisciplinary team working in primary care using normalisation process theory: protocol for an integrative review

Pauline O'Reilly, Siew Hwa Lee, Madeleine O'Sullivan, Walter Cullen, Catriona Kennedy, Anne MacFarlane

Citation

Pauline O'Reilly, Siew Hwa Lee, Madeleine O'Sullivan, Walter Cullen, Catriona Kennedy, Anne MacFarlane. Assessing the facilitators and barriers of interdisciplinary team working in primary care using normalisation process theory: protocol for an integrative review. PROSPERO 2015:CRD42015019362 Available from
http://www.crd.york.ac.uk/PROSPERO_REBRANDING/display_record.asp?ID=CRD42015019362

Review question(s)

The aim of this integrative review is to examine the evidence base relating to the team working processes of interdisciplinary primary care teams. The following questions formed the basis of the review:

1. What does the published literature tell us about the facilitators and barriers to interdisciplinary team working processes in formal, statutory primary care teams for the general population?

2. What, if any, are the main research gaps that require attention to support implementation?

Searches

We will search for published studies in the following databases: the Cochrane Library (Cochrane Database of Systematic Reviews, Cochrane Central Register of Controlled Trials (CENTRAL), Cochrane Methodology Register), MEDLINE, EMBASE, CINAHL, PsycINFO, AMED, ASSIA, TRIP, ISI Web of Science and Scopus.

Unpublished and in progress studies:

• Unpublished work (grey literature) which are not published in accessible

formats or indexed in the academic databases listed above. Examples

include conference proceedings.

• Hand searching articles from reference lists in retrieved articles will be

sourced to ensure completeness.

• On-going work and research in progress by searching the internet-based

relevant databases:

• www.who.int/ictrp/en/

• www.anzctr.org.au

• www.clinicaltrials.gov

• www.controlledtrials.com

Limits to include:

• Literature published in English Language only

• Publications from 2004 to 2014

Literature obtained from the search will be stored in EndNote Web, and referenced according to Harvard referencing style.

Types of study to be included
Quantitative and qualitative studies will be included.

We will exclude systematic reviews, discussion papers, opinion papers, non-empirical studies.

Condition or domain being studied
From an international perspective, team working in primary care is of paramount importance in the reform of primary care in order to provide cost effective and comprehensive care (World Health Organisation (WHO) 2008). Reforming primary care requires that the change process is acknowledged and that staff are supported during the process (Allan et al. 2015).

For the optimum development and functioning of interdisciplinary primary care teams, it is imperative to assess the factors that both promote and inhibit team working and to understand how these act as levers and barriers to the implementation process (O'Sullivan et al. 2015).

The proposed review is designed to do just that and it is innovative because it addresses a gap in national and international literature, employs a relevant theoretical framework to investigate reported practice about team working processes and therefore has the potential to generate findings that are transferable across healthcare jurisdictions. A contemporary social theory called Normalisation Process Theory (NPT) (May and Finch 2009) will be used. This theory is known to enhance understanding of implementation processes (McEvoy et al. 2014). The four constructs of the NPT relate to issues of 'sense making', 'enrolment', 'enactment', and 'appraisal'. The constructs will be used as a heuristic device to design a coding guide for both the extraction and analysis of data from the literature (Mair et al. 2012).

Participants/ population
Inclusion population:

• Members of statutory generalist primary care team (healthcare

professionals e.g. General Practitioners, Nurses, Physiotherapists,

Occupational therapists, Social Workers, administrators and managers

working as a team in a primary care setting)

Exclusion population:

• If the team is set up to work with a specialist group such as veterans'

health or a specific condition i.e. Mental Health Team

Intervention(s), exposure(s)
Inclusion:

• Team working (by team we mean a group of agents with a common goal

which can only be achieved by appropriate combinations of individual

activities)

Exclusion:

• Research studies relating to the education or training of

undergraduate/postgraduate healthcare professionals in interdisciplinary

team working

Comparator(s)/ control
All comparators/controls will be considered as part of the review.

Context
Studies will focus on primary care settings.

Outcome(s)
Primary outcomes
The integrative review will analyse and synthesise quantitative and qualitative evidence using the Normalisation Process Theory (NPT) constructs in identifying team working to inform:

• Sense making – How primary care professionals make sense of team

working?

• Enrolment – How they engage or 'buy in' within the team?

• Enactment – How they enact team working?

• Appraisal – How they appraise and reflect on team working?

Having a conceptual understanding, of how interdisciplinary teams work, will lead to the development of new knowledge to support implementation which will inform appraisal of the impact on health outcomes, within the primary care setting.

Secondary outcomes
None

Data extraction, (selection and coding)
Two reviewers will search the databases. The core team will then be divided into pairs and screen titles and abstracts for potentially relevant studies. Where necessary, any discrepancies will be resolved through consensus or arbitration involving a third reviewer. Full texts retrieved will be assessed against the inclusion criteria.

Two reviewers will independently extract and code data using the four constructs of the NPT theoretical framework viz, Sense-making, Engagement, Enactment, and Appraisal (May and Finch 2009).

Risk of bias (quality) assessment
Qualitative studies will be appraised using Noyes and Popay (2007) criteria. Quantitative studies will be appraised using the Scottish Intercollegiate Guidelines Network (SIGN) (2012) including a risk of bias assessment, where appropriate.

The overall quality of included studies will be evaluated using the SIGN grading system (2012).

Strategy for data synthesis
All coded data will be analysed using NPT constructs (May and Finch 2009) and synthesised following the principles of Framework Analysis (Ritchie and Spencer 1994).

Analysis of subgroups or subsets
Not applicable.

Dissemination plans

• The findings will inform policy and guidance including the Irish and international healthcare systems.

• To publish the findings in peer reviewed journals appropriate to the field.

• To present at national conference (e.g. Association of the University Departments of General Practice) and international academic primary care conferences e.g. Society for Academic Primary Care, UK.

Contact details for further information

Dr O'Reilly

Department of Nursing and Midwifery,

Faculty of Education and Health Sciences,

Health Sciences Building,

University of Limerick,

Limerick,

Ireland.

pauline.oreilly@ul.ie

Organisational affiliation of the review

University of Limerick, Ireland

http://www.ul.ie

Review team

Dr Pauline O'Reilly, Department of Nursing and Midwifery, Faculty of Education and Health Sciences, University of Limerick, Limerick, Ireland
Dr Siew Hwa Lee, Department of Nursing and Midwifery, Faculty of Education and Health Sciences, University of Limerick, Limerick, Ireland
Ms Madeleine O'Sullivan, Graduate Entry Medical School (GEMS) Faculty of Education and Health Sciences, University of Limerick, Limerick, Ireland
Professor Walter Cullen, School of Medicine and Medical Science, University College Dublin, Ireland
Professor Catriona Kennedy, Department of Nursing and Midwifery, Faculty of Education and Health Sciences, University of Limerick, Limerick, Ireland
Professor Anne MacFarlane, Graduate Entry Medical School (GEMS) Faculty of Education and Health Sciences, University of Limerick, Limerick, Ireland

Details of any existing review of the same topic by the same authors

Not applicable.

Anticipated or actual start date

01 September 2014

Anticipated completion date

31 December 2015

Funding sources/sponsors
None

Conflicts of interest
None known

Other registration details
None

Language
English

Country
Ireland

Subject index terms status
Subject indexing assigned by CRD

Subject index terms
Humans; Interdisciplinary Studies; Primary Health Care

Stage of review
Ongoing

Date of registration in PROSPERO
15 July 2015

Date of publication of this revision
15 July 2015

DOI
10.15124/CRD42015019362

Stage of review at time of this submission	Started	Completed
Preliminary searches	Yes	Yes
Piloting of the study selection process	Yes	Yes
Formal screening of search results against eligibility criteria	Yes	No
Data extraction	No	No
Risk of bias (quality) assessment	No	No
Data analysis	No	No

PROSPERO
International prospective register of systematic reviews
The information in this record has been provided by the named contact for this review. CRD has accepted this information in good faith and registered the review in PROSPERO. CRD bears no responsibility or liability for the content of this registration record, any associated files or external websites.
